# Toward Building Hybrid Biological/*in silico* Neural Networks for Motor Neuroprosthetic Control

**DOI:** 10.3389/fnbot.2015.00008

**Published:** 2015-08-11

**Authors:** Mehmet Kocaturk, Halil Ozcan Gulcur, Resit Canbeyli

**Affiliations:** ^1^Institute of Biomedical Engineering, Bogazici University, Istanbul, Turkey; ^2^Department of Biomedical Engineering, Istanbul Medipol University, Istanbul, Turkey; ^3^Department of Psychology, Bogazici University, Istanbul, Turkey

**Keywords:** neuroprosthetics, brain–machine interface, motor cortex, spiking neuron models, spike timing-dependent plasticity

## Abstract

In this article, we introduce the Bioinspired Neuroprosthetic Design Environment (BNDE) as a practical platform for the development of novel brain–machine interface (BMI) controllers, which are based on spiking model neurons. We built the BNDE around a hard real-time system so that it is capable of creating simulated synapses from extracellularly recorded neurons to model neurons. In order to evaluate the practicality of the BNDE for neuroprosthetic control experiments, a novel, adaptive BMI controller was developed and tested using real-time closed-loop simulations. The present controller consists of two *in silico* medium spiny neurons, which receive simulated synaptic inputs from recorded motor cortical neurons. In the closed-loop simulations, the recordings from the cortical neurons were imitated using an external, hardware-based neural signal synthesizer. By implementing a reward-modulated spike timing-dependent plasticity rule, the controller achieved perfect target reach accuracy for a two-target reaching task in one-dimensional space. The BNDE combines the flexibility of software-based spiking neural network (SNN) simulations with powerful online data visualization tools and is a low-cost, PC-based, and all-in-one solution for developing neurally inspired BMI controllers. We believe that the BNDE is the first implementation, which is capable of creating hybrid biological/*in silico* neural networks for motor neuroprosthetic control and utilizes multiple CPU cores for computationally intensive real-time SNN simulations.

## Introduction

**Figure 1:**
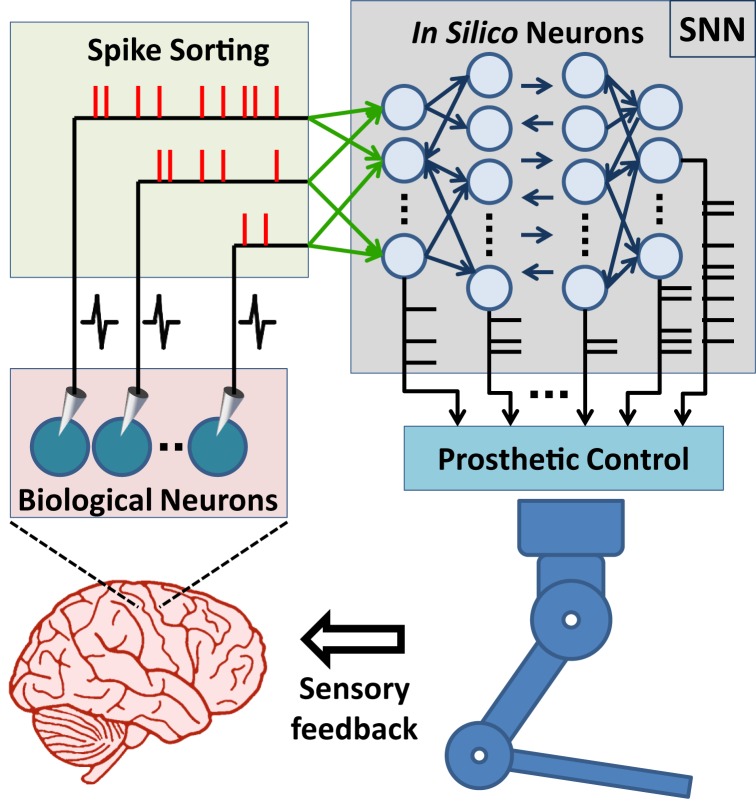
**Principal components of SNN-based neuroprosthetic control paradigm**. A spike sorting utility continuously acquires neural signals from the brain and extracts the timestamps of the spikes generated by real neurons. The spike events are then streamed to the biologically plausible model neurons as “virtual” synaptic inputs and these inputs trigger further information processing in the spiking neural network (SNN). Finally, the prosthetic control module monitors the spike event outputs of the SNN and translates them into prosthetic command signals for manipulation of the neuroprosthesis.

Advances in brain–machine interface (BMI) technologies have allowed rodents (Chapin et al., 1999; DiGiovanna et al., 2009; Manohar et al., 2012), monkeys (Taylor et al., 2002; Carmena et al., 2003; Velliste et al., 2008; Dethier et al., 2011; Pohlmeyer et al., 2014), and humans (Hochberg et al., 2006, 2012; Collinger et al., 2013; Wodlinger et al., 2015) to control different prosthetic devices directly with their neuronal activity. In conventional BMI design approach, the main motivation has generally been to find an input–output mathematical model, which optimally transforms *firing rates* of cortical neurons into control signals for manipulation of a prosthetic actuator. In these systems, spike binning is performed in order to provide firing-rate inputs to the model used and this process leads to loss in the information encoded by the timing of neural spikes (Riehle, 1997; Hatsopoulos et al., 1998; Grammont and Riehle, 2003; Engelhard et al., 2013). Based on an *input-output model* or *transform*, information processing principles of these systems are fundamentally different from those of natural neural circuits (Carmena, 2013). From a more biologically inspired design perspective, the BMI controllers could be formed using a spiking neural network (SNN). In such a control paradigm, the SNN would consist of biologically plausible model neurons and receive simulated synaptic inputs from the extracellularly recorded cortical neurons. The controller therefore would form a hybrid biological/*in silico* neural network with the neuronal circuits of the user’s brain. Its outputs would then be used in manipulating a neuroprosthesis (Figure 1). This novel, SNN-based design approach has the potential to bring several advantages in neuroprosthetic system control, adaptation, and implementation. First, the information encoded by spike timing could be used at the input layer of the SNN-based BMI controller. Second, spike timing plays a critical role in neuroplasticity (Markram et al., 2011), which is essential in neuroprosthetic learning (Koralek et al., 2012). Therefore, the SNN-based BMI controllers updating their parameters by simulating mechanisms of spike timing-dependent plasticity might have superior adaptation performance than existing firing rate-based neuroprosthetic systems. Third, implementation of the SNN-based BMI controllers into neuromorphic chips (Dethier et al., 2011; Indiveri et al., 2011) can enable delivery of fully implantable, ultra low-power neuroprosthetic systems for paralyzed patients. In addition, the SNN-based design approach can also be beneficial in the field of neuroscience. The interactions of real neurons with model neurons could be investigated during neuroprosthetic control experiments and these investigations can provide new insights into the information processing principles in the motor cortex during neuroprosthetic control and learning.

While offering to provide promising methods for neuroscience and BMI research, development process of SNN-based neuroprosthetic controllers requires powerful and purpose-specific platforms, which are capable of (1) real-time SNN simulation, (2) providing biologically realistic synaptic interactions between real and model neurons, and (3) manipulating a robotic actuator according to the spike outputs of the model neurons in real-time. There are advanced software projects, such as RTXI (Lin et al., 2010; Kispersky et al., 2011) and RELACS (Grewe et al., 2011), for dynamic clamp experiments (Dorval et al., 2007), where a *hybrid* neural network is created by bidirectionally interfacing one or a few neurons with one or several model neurons through simulated synapses using *intracellular* recording and stimulation techniques. Moreover, NeuroRighter also provides practical tools for extracellular recordings and closed-loop stimulation of neural circuits in real-time (Rolston et al., 2010; Newman et al., 2012). However, these systems have not been specifically developed for creating simulated synaptic connections between a SNN and tens of extracellularly recorded neurons for real-time control of a robotic arm. Toward addressing the listed requirements of the SNN-based neuroprosthetic control paradigm, we implemented the “Bioinspired Neuroprosthetic Design Environment (BNDE).” The BNDE supports extracellular recording from 32 data acquisition (DAQ) channels and efficiently utilizes multiple CPU cores for computationally intensive real-time SNN simulations. It realizes a real-time interface between a SNN and a motor neuroprosthesis. In this paper, we introduce the BNDE’s hardware and software techniques.

In the present article, we also demonstrate, on the BNDE, how to develop a novel, adaptive bioinspired BMI (B-BMI) control algorithm, which combines a reward-modulated spike timing-dependent plasticity rule with a winner-take-all type classifier for one-dimensional control of a robotic arm and discuss its performance using real-time closed-loop simulations. Besides benefiting from the potential advantages of SNN-based neuroprosthetic control paradigm, which are listed above, the present B-BMI performs adaptation by simulating a possible mechanism of dopamine-dependent spike timing-dependent plasticity. A reward signal, which may correspond to phasic changes in dopamine concentration in natural neural circuits, is used to update synaptic weight parameters of the B-BMI. If the reward signal presented in the control architecture of the B-BMI can be extracted directly from the dopaminergic activity of the user’s brain during neuroprosthetic learning, this system can update its parameters without an external training signal (Mahmoudi et al., 2013; Marsh et al., 2015). The computational capacity of the BNDE has also been evaluated using a stress test paradigm for more sophisticated future BMI controller applications with higher number of model neurons and parameters.

## Materials and Methods

### The bioinspired neuroprosthetic design environment

**Figure 2:**
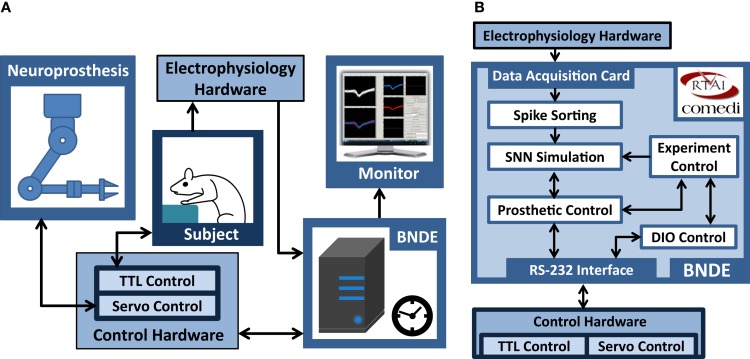
**Generic hardware and software components of the BNDE**. **(A)** The hardware components. The BNDE is implemented around a desktop PC. Electrophysiology hardware provides analog neural signal inputs to the data acquisition card. The control hardware drives the servomotors of the neuroprosthesis and binary-stated components (e.g., LEDs, levers) of the experimental environment, and communicates with the BNDE through RS-232 interface. **(B)** The real-time (RT) tasks of the BNDE running on the RTAI-equipped PC. The information flow directions between RT tasks and hardware components are shown using arrows.

The design philosophy of the BNDE aims to combine the flexibility of software-based real-time signal processors and SNN simulators with powerful hardware resources around a standalone desktop PC (Figure 2). To achieve real-time performance for the tasks involved in SNN-based neuroprosthetic control, the BNDE was developed around a quad-core PC equipped with Real-time Application Interface (RTAI)^1^.

RTAI is an open-source, hard real-time extension for Linux operating system (Mantegazza et al., 2000); it guarantees strict timing constraints of real-time applications while also allowing execution of standard Linux features and services (e.g., window system, keyboard/mouse inputs, file system, Linux applications, etc.) on the same system. In order to achieve this, RTAI handles the Linux operating system as a lowest priority task and enables (highest priority) real-time (RT) tasks to preempt Linux services whenever needed. As a consequence, no unexpected delays or interruptions occur in execution of the RT tasks. From the point of the user, working of the system remains the same as in a standard Linux operating system since standard Linux services are still allowed to run on the system when no RT task is executing (Mantegazza et al., 2000). Based on this feature of RTAI, we implemented the BNDE as a practical solution for the development process of neurally inspired BMI controllers; it has been equipped with both RT tasks and non-time-critical applications. While the RT tasks guarantees the timing constraints for biological–*in silico* neuronal interactions through simulated synapses and real-time SNN simulations, the non-time-critical applications enable live visualization of experimental data and execution of graphical user interfaces (GUIs) for management of the behavioral experiments. Thus, the experimenter is able to monitor the spiking activity patterns and dynamics of the simulated neurons online while the RT tasks are performing the time-critical operations in the background.

As illustrated in Figure 2, the BNDE mainly executes five RT tasks, which present a framework for implementation of SNN-based BMIs and behavioral paradigms for *in vivo* studies. These tasks are as follows: (1) spike sorting, (2) SNN simulation, (3) prosthetic control, (4) digital input–output (DIO) control, and (5) experiment control tasks. In this section, we briefly explain the role of these tasks; more detailed explanation about how each task works will be given in Section “Real-Time Closed-Loop Simulation Methods” by demonstrating the implementation of a SNN-based BMI.

*The spike sorting task* of the BNDE works in conjunction with a DAQ device to acquire and process the analog neural signals provided by a standard extracellular neural recording system consisting of microelectrode assemblies, signal filters, and amplifiers (Nicolelis et al., 1997). In the BNDE, the DAQ device (National Instruments, PCIe-6259) is configured to perform continuous analog-to-digital conversion (ADC) with its maximum sampling rate of 31.25 kHz per channel and the spike sorting task executes the signal processing routines for extracting single-unit spikes through the neural data provided by the DAQ device. For each detected single-unit spike, the spike sorting task delivers an event to the post-synaptic neurons of the SNN to provide simulated synaptic interactions between biological and *in silico* neurons.

*The SNN simulation task* executes every 2 ms and performs numerical integrations to evaluate the dynamics of the neurons in the SNN. In the present implementation of the BNDE, there are two SNN simulation tasks each of which is assigned to a different CPU core (Table 1) and simulates half the neurons in the SNN. Thus, it becomes possible to allocate two cores of the CPU for computationally intensive SNN simulations. For implementation of the SNN, we utilize Izhikevich’s simple neuron model, which is capable of exhibiting the rich dynamic repertoire of real neurons with simple differential equations (Izhikevich, 2003, 2004, 2007b).

**Table 1:** **The RT tasks of the BNDE and CPU core assignments**.

RT task	CPU core	Task period (TP)
Spike sorting	0	512 μs
SNN simulation	1 and 2	2 ms
Prosthetic control	3	2 ms
Experiment control	3	2 ms
DIO control	3	2 ms

*The prosthetic control* and *the DIO (digital input–output) control tasks* operate in cooperation with the in-house built, microcontroller-based (Microchip, PIC18F4520) *control hardware*, which enables the control of the experimental environment components by its embedded software modules [i.e., the *servo control module* and the *Transistor–Transistor Logic (TTL) control module*]. To achieve the communication with the *control hardware*, the BNDE utilizes a commercially available generic RS-232 interface controller which transmits and receives data at 115.2 kbd/s.

*The prosthetic control task* is the intermediary between SNN simulation tasks and the *servo control module* of the *control hardware*. It buffers the spike events received from the output layer of the SNN and translates them into pulse width commands to be handled by the servo control module. The servo control module then drives the three actuators or joints of a customized version of Lynxmotion AL5D robotic arm (Swanton, VT, USA) and returns the angle values of the joints through the same interface. By receiving the joint angles of the robotic arm, the prosthetic control task calculates the Cartesian position of the tip of the arm by forward kinematics.

*The DIO control task* receives the status of the digital inputs from experimental environment through the *TTL control module* of the *control hardware* and determines if the time to trigger an event is expired for a digital input. For instance, if a lever is pressed for a certain amount of time, it can trigger an event (successful lever press) and send it to *experiment control task* to request a trial initiation. In addition, it delivers digital outputs to the *TTL control module* to alter the binary state of the experimental environment components (e.g., turn LED off, release reward, etc.).

*The experiment control task* is the management center of the behavioral experiments. By receiving messages from the prosthetic and DIO control tasks, it decides if a trial should be initiated, ended, rewarded, etc. In addition, it informs the downstream tasks (i.e., SNN simulation, DIO control, and prosthetic control tasks) about the decisions it made so that they take action to apply the requirements of the experimental paradigm.

To utilize the system resources efficiently, the RT tasks of each module are assigned to run on a particular core of the CPU (Intel i7-950, Table 1).

In order to facilitate the SNN-based neuroprosthetic control studies, the BNDE was also equipped with non-time-critical applications, which provide GUIs and online data visualization tools easing the management process of the experiments. For the implementation of the GUIs, we utilized open-source GTK+libraries^2^. In addition, using the GtkDatabox libraries (sourceforge.net/projects/gtkdatabox), we implemented online data visualization utilities, which provide live display of the continuously changing signals (e.g., neural signals, dynamics of *in silico* neurons, neuronal spike trains, etc.).

The GUI for the spike sorting process enables the configuration of the DAQ device and visualization of the acquired neural signals through a software-based oscilloscope. It also provides visualization tools for determination of the thresholds and templates for spike sorting process. The GUI related to the SNN simulation allows the user to build a neural network consisting of spiking neurons and visualize the dynamics of the neurons and spiking activity patterns of the real neurons during the behavioral experiments. The other GUIs provide the software forms for adjustment of the parameters related to the management of the experiments (e.g., for submitting the maximum duration of a trial, the length of a valid lever press, etc.) and handle online data recording utilities, which periodically save experimental data to the hard drive of the system.

All software code for the BNDE and its libraries has been developed using C programing language. The libraries contain the routines for (1) creating and executing live data visualization tools, (2) building hybrid neural networks consisting of real neurons, which are extracellularly recorded, and model neurons, which are described using Izhikevich’s biologically plausible model (Izhikevich, 2007b), (3) communicating with the control hardware, (4) manipulating the three degree-of-freedom Lynxmotion AL5D robotic arm and calculating the Cartesian position of its endpoint by forward kinematics, (5) controlling the binary-stated experimental environment component, such as levers and LEDs, and (6) data recording. The libraries also include templates for messaging between the RT tasks. The messaging between the RT tasks of the BNDE is performed using the shared memory feature of the RTAI.

The BNDE has been developed using Ubuntu Linux 10.04 LTS with kernel v2.6.32.2 and RTAI v3.8. In the current implementation, the COMEDI drivers with version 0.7.76 have been utilized for the DAQ card. GTK+ libraries with version 2.0 and GtkDatabox libraries with version 0.9.1.1 have been used for implementation of GUIs and online visualization tools.

#### Real-Time Biological/*In Silico* Neuronal Interaction in the Hybrid Neural Network

In order to acquire the neural signals and provide biologically realistic synaptic interactions between real and *in silico* neurons in the SNN-based neuroprosthetic control paradigm, we utilized the open-source DAQ card drivers provided by the COMEDI (Linux Control and Measurement Device Interface)^3^ project and the DAQ-related APIs provided by the RTAI developers^1^. Using these drivers and APIs, it becomes possible to bypass the interrupt management layers of the standard Linux kernel (Mantegazza et al., 2000) and provide deterministic responses to the hardware interrupts of the DAQ device; a high priority RT task can be immediately executed whenever the DAQ device generates an interrupt.

**Figure 3:**
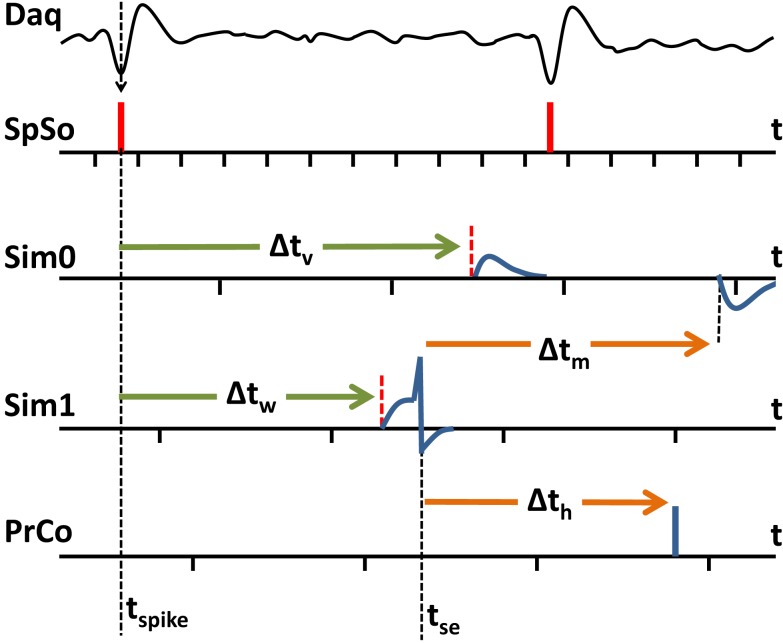
**An example of spike event delivery between the tasks of the BNDE**. SpSo: spike sorting task, Sim0 and Sim1: SNN simulation tasks, PrCo: prosthetic control task. The execution times of tasks are shown by the ticks on time axes. The tasks are periodically triggered. Task period for SpSo is 512 μs. The task periods for Sim0, Sim1, and PrCo are 2 ms as also shown in Table 1. In the present example, a neural spike time (*t*_spike_) is determined through the extracellular recordings according to the lowest peak of the spike waveform. A synaptic event for this spike is scheduled and delivered to the post-synaptic *in silico* neurons to be handled at *t*_spike_ + Δ*t*_v_ and *t*_spike_ + Δ*t*_w_. After being depolarized, the neuron simulated by Sim1, but not the one simulated by Sim0, generates a spike at *t*_se_ and then events are scheduled for *t*_se_ + Δ*t*_m_ and *t*_se_ + Δ*t*_h_ to be handled by Sim0 and PrCo, respectively. Since the neuron simulated by Sim1 is inhibitory, it leads to hyperpolarization in the post-synaptic neuron simulated by Sim0. In the BNDE, whenever an event is generated, it is immediately transmitted to the target task by event scheduling. The scheduled event is then handled by the receiver task in the corresponding execution cycle. The value of an event transmission delay (Δ*t*_v_, Δ*t*_w_, Δ*t*_m_, Δ*t*_h_) should not be smaller than the period of the task delivering the event so that the target task guarantees not to miss any scheduled event. The period of the tasks can be adjusted by the user according to the event transmission delay requirements of the BMI to be designed.

In the BNDE, the 32-channel DAQ device is configured to generate an interrupt subsequent to acquiring 16 samples for each channel and the spike sorting task is configured to execute in response to the interrupts of the DAQ device. The sampling rate of the DAQ device is set to 31.25 kHz per channel. Therefore, the spike sorting task runs every 512 μs. Whenever the spike sorting task runs, it (1) writes acquired samples into a separate circular buffer for each DAQ channel, (2) filters the buffered neural signals by a fourth order Butterworth digital band-pass filter (cut-off frequency = 400 Hz–8 kHz), (3) up-samples the filtered neural data to 62.5 kHz by cubic interpolation to improve spike alignment performance for spike sorting, (4) detects the neural spikes by level thresholding. In the BNDE, a spike waveform is represented by 18 DAQ samples. Whenever the spike sorting task retrieves 18 samples for a detected spike waveform, it (1) runs template matching algorithm for sorting (as explained in Section “Spike Sorting via Template Matching”), (2) timestamps the sorted spike according to the lowest peak of its waveform, and (3) schedules synaptic events for the corresponding post-synaptic *in silico* targets (Figure 3).

The following algorithm briefly explains the routines of the spike sorting task. These routines also realize synchronization between the clocks of the DAQ hardware and the spike sorting task.

1. Initialize DAQ card and read system time.
  *t*_previous_ = rt_get_time().
2. Wait for DAQ interrupt (generated every 512 μs).
3. Read system time.
  *t*_current_ = rt_get_time();
4. Perform clock synchronization.
  *t*_expected_ = *t*_previous_ + 512 μs.
  if *t*_expected_ > *t*_current_
  then *t*_current_ = *t*_current_ + Δ*t*_sync_
  else *t*_current_ = *t*_current_ − Δ*t*_sync_
5. Buffer all acquired (512) samples, perform digital filtering and interpolation for all 32 channels.
6. Perform spike sorting for GUI-activated single units.
7. Timestamp sorted spikes according to *t*_current_ and schedule synaptic events for *in silico* neurons.
8. Save system time for next acquisition.
  *t*_previous_ = *t*_current_;
9. Repeat step 2.

In the above algorithm, we set Δ*t*_sync_ to 0.5 μs so that the clock synchronization is achieved in the BNDE. In this algorithm, *t*_current_ follows *t*_expected_ with small synchronization steps.

For synaptic interactions in the hybrid neural network, each post-synaptic *in silico* neuron has a *separate* circular buffer for each incoming synapse. As a result of this buffering mechanism, the spike sorting task and multiple SNN simulation tasks, which are run on different CPU cores, can *simultaneously* deliver scheduled synaptic events to any post-synaptic neuron. Therefore, no mutual exclusion lock, which suspends all CPU cores until unlocking, is used in synaptic event buffering mechanisms and the computational resources of the system are efficiently used for real-time SNN simulations.

In the BNDE, whenever a pre-synaptic (real or *in silico*) neuron fires, it schedules a synaptic event and writes this event into the corresponding synaptic event buffer of the post-synaptic neuron. At the beginning of each integration step, the post-synaptic neuron reads all its synaptic event buffers, sorts the events in the time domain in a separate buffer and performs integration by reading this buffer. Consequently, biologically realistic synaptic interactions between real and *in silico* neurons are realized. More details about the integration methods for simulating the SNN will be given in Section “Real-Time Closed-Loop Simulation Methods” by demonstrating the implementation of a SNN-based BMI controller on the BNDE.

#### Spike Sorting via Template Matching

In the BNDE, each single-unit, which is sorted from a recording channel, is modeled with a multivariate Gaussian distribution, N(μ, Σ). The likelihood of a detected spike waveform given a particular single-unit or *class* C*_i_* is given (Lewicki, 1998; Alpaydin, 2010):

(1) $$p(x|C_{\text{i}})=1/({\left(2\text{π}\right)}^{\left(\text{d}/2\right)}\left|\Sigma_{\text{i}}{|}^{\left(1/2\right)}\right)\;\exp[-1/2{\left(x-{\text{μ}}_{\text{i}}\right)}^{\text{T}}\;\Sigma_{\text{i}}{\;}^{\left(-1\right)}(x-{\text{μ}}_{\text{i}})]$$

where *x* is the *d*-dimensional spike waveform data vector (*d* = 18 samples), μ_i_ and Σ_i_ are mean and covariance matrix for unit C*_i_*, respectively.

**Figure 4:**
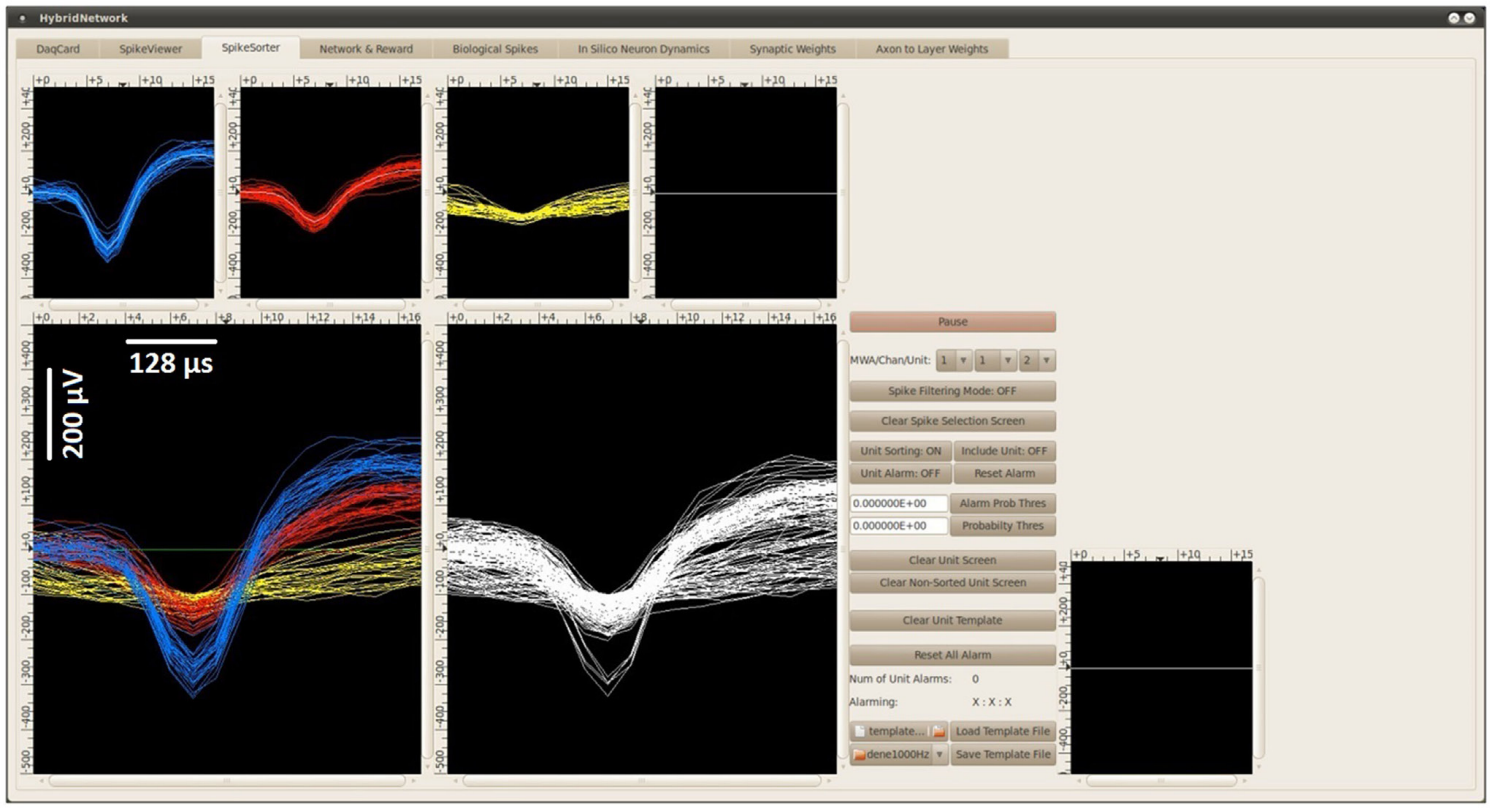
**The spike sorting GUI of the BNDE displaying the spikes recorded from an awake rat, which was chronically implanted with a platinum/iridium microwire array in the forelimb area of the M1**. Each spike in the BNDE is represented by 18 DAQ samples; therefore, the span of a spike waveform is 576 μs. In the present example, two single-units were well-isolated from one channel of the *in vivo* recordings. The spike waveforms of these units are shown by “blue” and “red” colors. The third unit, whose waveforms are shown by “yellow” color, is used to define noise or low-amplitude spikes which are not suitable to be used in neuroprosthetic control. Each spike, whose amplitude exceeds a manually determined threshold, is sorted into one of these units appropriately by template matching as explained in Section “Spike Sorting via Template Matching.” The spike sorting GUI supports selection of (well-isolated) single-units, which can be connected to the *in silico* neurons of the BMI controller through simulated synapses.

In the BNDE, the thresholds for spike detection and templates for spike sorting are manually determined by the experimenter using the GUIs implemented for these purposes. The thresholds are set using a software-based oscilloscope, which displays the acquired neural signal from a selected DAQ channel. When a spike detection threshold for a recording channel is set, the spike sorting *GUI* of the BNDE (Figure 4) starts to display the spike waveforms acquired from that channel online. Using the mouse cursor over the spike sorting GUI, the experimenter selects the spike waveforms belonging to a single-unit. Based on 60 selected spike waveforms, the spike sorting GUI forms a *template* for 1 single-unit, described by μ_i_ and Σ_i_. The spike sorting task, runs every 512 μs, then applies the likelihood function (Eq. 1) for each detected spike for each single-unit or class and sorts the detected spike into the single-unit (C*_i_*) to which it has the highest probability of belonging. Whenever a spike is sorted, the spike sorting task schedules synaptic events for the corresponding post-synaptic *in silico* cells (Figure 3). Moreover, the spike sorting GUI displays the sorted spike waveform with a color code corresponding to that unit (Figure 4). As an aside, μ_i_, $\sum_{\text{i}}^{-1}$, and $|\Sigma_{\text{i}}{|}^{\frac{1}{2}}$, utilized in Eq. 1, are evaluated by the spike sorting GUI only once when the spike waveforms are selected for forming the templates. In this way, it becomes computationally practical for the spike sorting task to apply the likelihood function (Eq. 1) for each detected spike.

Using the spike sorting GUI of the BNDE, a probability threshold for each isolated unit is submitted by the user so that an interfering signal, whose probability of belonging to any single-unit is very low, are discarded from sorting. The waveforms of the detected interfering signals are plotted by the spike sorting GUI. Using the same GUI, all information related to the spike templates and thresholds are saved into a file to be used in later experiments.

### The bioinspired brain–machine interface controller

In this section, we describe the B-BMI controller, which is based on building a hybrid biological/*in silico* neural network consisting of extracellularly recorded motor cortex neurons and model medium spiny neurons (MSNs).

The MSNs are inhibitory projection neurons and comprise the majority of the neuronal population in the striatum (approximately 95% in the rat) (Tepper et al., 2007). They receive excitatory (glutamatergic) synaptic inputs from the cortical areas and dopaminergic inputs from the midbrain. The dopaminergic inputs, which may encode the difference between predicted and received rewards (namely *reward prediction error*) by biphasic activity changes (Schultz, 1998), modulate plasticity in the synapses between the cortical neurons and the MSNs (Reynolds and Wickens, 2002; Wickens et al., 2003; Kreitzer and Malenka, 2008; Pawlak and Kerr, 2008).

The MSNs have unusual, bistable membrane potential properties. They have a high threshold for activation and very low activity profile during resting conditions (down-state). When they are depolarized by strong excitatory inputs, their membrane potentials remain in “up-state” for a prolonged period. In the up-state, the membrane potential is close to firing threshold and generation of a spike (Grillner et al., 2005; Izhikevich, 2007b).

**Figure 5:**
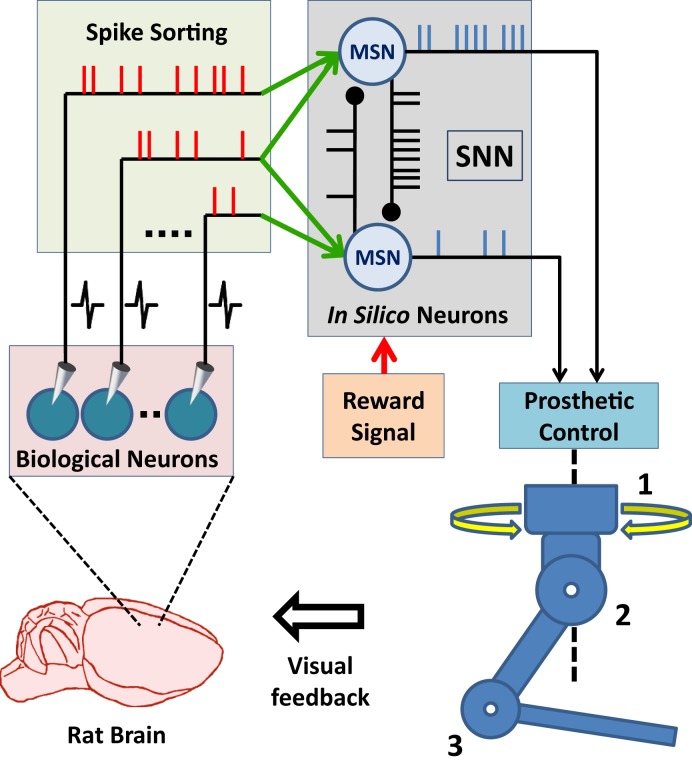
**Control architecture of the Bioinspired BMI (B-BMI)**. The SNN consists of two MSNs, which receive simulated excitatory synaptic inputs from the extracellularly recorded motor cortex units. The MSNs are reciprocally connected to each other through inhibitory synapses. The prosthetic control module monitors the spiking activity of the MSNs and determines the one with the highest firing rate as the winning neuron. The action corresponding to the winning neuron is applied by the digital base servomotor (Joint 1) of the robotic arm in one-dimensional space. A global reward signal modulates the weights of the excitatory synapses between the motor cortex and medium spiny neurons through a reward-modulated spike-timing-dependent plasticity rule.

**Figure 6:**
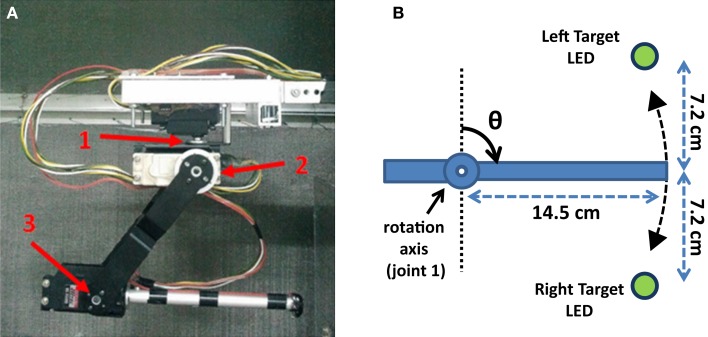
**The customized Lynxmotion AL5D robotic arm controlled by the B-BMI in a two-target reaching task in one-dimensional space in real-time**. **(A)** Side view and joints of the robotic arm. The joints of the three degrees-of-freedom robotic arm are pointed using red arrows. **(B)** The representative top view of the robotic arm in closed-loop control. Two opposite left/right targets, pointed by green LEDs, were located on the same plane to be reached by the robotic arm moving around its first joint (base servomotor) in one-dimensional space.

By utilizing the knowledge related to bistable membrane potential properties and dopamine-dependent plasticity of MSNs, we created the B-BMI controller (B-BMI) to realize a two-target center-out reaching task in one-dimensional space (Figures 5 and 6). In the control paradigm, the living (extracellularly recorded) primary motor cortex (M1) units are partially connected to the *in silico* MSNs through simulated excitatory synapses. The MSNs are then reciprocally connected through strong inhibitory synapses to build a mechanism for winner-take-all competition. In the present control paradigm, the MSN with the highest spike count is selected as the winning neuron and the prosthetic action (moving to the “left” or “right”) corresponding to that neuron is applied by the base servomotor of the robotic arm in one-dimensional space. In case of equality among the spike counts of the MSNs, no winning neuron is selected and no prosthetic action is applied (“stationary”). The spike counts of the MSNs are calculated by binning the generated spikes every 26 ms with a sliding 104 ms time window.

The *in silico* MSNs of the B-BMI are described using the equations of Izhikevich’s simple neuron model (Izhikevich, 2007b) and the synaptic interactions are provided by fast conductance-based synaptic currents (Vogels and Abbott, 2005) as in Stewart and Bair (2009):

(2) $$Cv\prime =k\left(v-v_{\text{r}}\right)\left(v-v_{\text{t}}\right)-u-\eta \left(v-E_{\eta }\right)-\gamma \left(v-E_{\gamma }\right)$$

(3) u′=a(bv−u)

where ν is membrane potential, *u* is membrane recovery variable, ν_r_ is the resting membrane potential, ν_t_ is the threshold potential, *C* is membrane capacitance, *a* is a constant, which describes time scale of *u*, *b* is a constant, which describes the sensitivity of *u*, *k* is a scaling constant, η and γ are total excitatory and inhibitory synaptic conductances, respectively. *E*_η_ and *E*γ represent excitatory and inhibitory synaptic reversal potentials. Arrival of a synaptic event from biological or *in silico* pre-synaptic neuron leads to a step-wise increase in the appropriate conductance variable; η → η + *w*_i_ for an excitatory event and γ → γ + *w*_i_ for an inhibitory event, where *w*_i_ is the conductance value or “weight” of the *i*-th synapse of the neuron. When there is no incoming event, the total conductance values decay with time constants τ_η_ and τγ:

(4) $$\eta \prime =-\eta /\tau_{\eta }$$

(5) $$\gamma \prime =-\gamma /\tau_{\gamma }$$

When the membrane potential exceeds a voltage peak (ν_peak_), i.e., the neuron generates a spike, the membrane potential and membrane recovery variable are reset as follows:

(6) $$\;v\geq v_{\text{peak}}\text{ then }\left\{ \begin{array}{l} v\leftarrow c\\ u\leftarrow u+d \end{array}\right.$$

The neuron model parameters for the MSNs are *a* = 0.01, *b* = −20, *c* = −55 mV, *d* = 150, *C* = 50 pF, *k* = 1, ν_r_ = −80 mV, ν_t_ = −25 mV, ν_peak_ = 40 mV (Izhikevich, 2007b). Through a number of trial-and-error studies to implement an effective winner-take-all operation in the system, the reversal potentials and the time constants for conductance values were set as follows: *E*_η_ = 0 mV, E_γ_ = −110 mV, τ_η_ = 6 ms, and τ_γ_ = 20 ms. The synaptic delays between the M1 units and the MSNs are selected from a uniform distribution between 3 and 5 ms and the delays between MSNs are selected from a uniform distribution ranging from 2.5 to 3.0 ms.

Learning in the network is provided by reward-modulated spike-timing-dependent plasticity (STDP), where a global reward signal leads to long-term potentiation (LTP) or depression (LTD) in the excitatory synapses (Frémaux et al., 2010). The initial weights of the synapses are given equally and the weight (*w*_ij_) of the synapse between the *i*-th motor cortex unit and the *j*-th MSN is updated every 26 ms as follows:

(7) $$w_{\text{ij}}\left(t+1\right)=w_{\text{ij}}\left(t\right)+\Delta w_{\text{ij}}$$

(8) $$\Delta w_{\text{ij}}=\mu w_{\text{ij}}\left(t\right)r\left(t\right)e_{\text{ij}}(t)$$

where μ is the learning rate, *e*_ij_(*t*) is the binary-stated (0 or 1) eligibility trace, which is triggered when the post-synaptic node *j* fires after the pre-synaptic node *i* within a time window of 40 ms and is terminated 100 ms after being triggered (Izhikevich, 2007a; Chadderdon et al., 2012; Dura-Bernal et al., 2014; Neymotin et al., 2013). *r*(*t*) is the current global reward signal evaluated as follows:

(9) $$r\left(t\right)=(1-{\bar{R}}_{\text{k}})S(t)$$

where *S*(*t*) is the sensory error (−1 or +1), which represents the consistency or discrepancy between the user’s expected movement direction and the actual robotic action. The sensory error is extracted from the movements of the robotic actuator every 26 ms and determines the sign of the global reward signal *r*(*t*). If the tip of the robot moves toward the currently selected target, the value of *S*(*t*) is 1, otherwise −1. ${\bar{R}}_{\text{k}}$ is the positive reward estimate (successful target reach estimate) for the k-th target and is updated at the end of each trial as a running mean (Vasilaki et al., 2009):

(10) $${\bar{R}}_{\text{k}}\left(n_{\text{k}}\right)=\left(1-\frac{1}{m}\right){\bar{R}}_{\text{k}}\left(n_{\text{k}}-1\right)+\frac{1}{m}R_{\text{T}}$$

where *n*_k_ is the trial number for the corresponding k-th target, *R*_T_ is the binary reward variable which indicates if the trial is ended with successful target reach or not (1 or 0), and *m* is the width of the averaging window.

After updating all synaptic weights using Eq. 7, a “homeostatic synaptic plasticity” rule (Turrigiano, 1999; Abbott and Nelson, 2000; Royer and Paré, 2003) is utilized to stabilize the excitability of the MSNs; the excitatory synaptic weights are normalized so that the sum of all weights of excitatory synapses to the *j*-th MSN is kept at a constant value *W*:

(11) $$w_{\text{ij}}\left(t+1\right)=\frac{w_{\text{ij}}\left(t+1\right)}{\sum_{\text{i}}w_{\text{ij}}\left(t+1\right)}W$$

In addition, the weight of the excitatory synapses is limited by a maximum value (*w*_max_) in order to avoid excessive increase in a synaptic weight. The value of *w*_max_ is determined as follows:

(12) $$w_{\text{max}}=\alpha (W/N_{\text{j}})$$

where *N*_j_ is the total number of excitatory synapses to the *j*-th MSN. α is a scaling constant which is >1 and determines the amount of the difference between *w*_max_ and the average of the weights of the excitatory synapses to the *j*-th MSN.

### Real-time closed-loop simulation methods

To evaluate the practicality of the BNDE for neuroprosthetic control experiments and study the performance of the B-BMI, we performed real-time closed-loop simulations, which involved a behavioral paradigm and an external, hardware-based neural signal synthesizer. The neural signal synthesizer and the behavioral paradigm were designed to realize a full system test in which all the software and hardware modules of the BNDE are utilized. The behavioral paradigm involves external binary inputs (e.g., button press) to initiate neuroprosthetic control trials and binary outputs to indicate the position of the targets (e.g., LED targets, Figure 6B) to be reached. Additionally, the neural signal synthesizer provides analog signals to the DAQ device of the system to imitate the extracellular recordings of *in vivo* experiments. The present closed-loop simulation paradigm also demonstrates an example of how to develop a SNN-based BMI controller using the BNDE.

**Figure 7:**
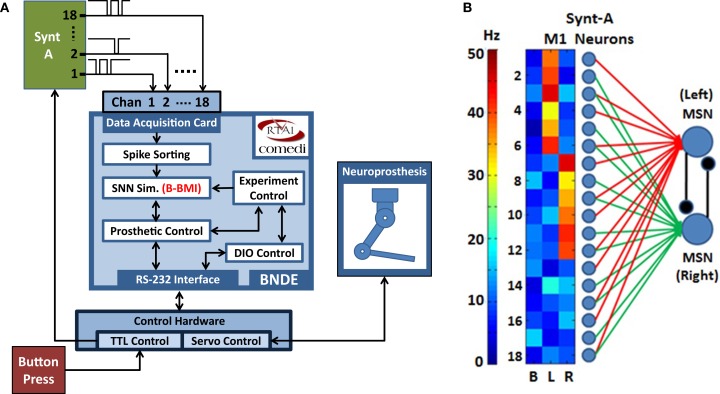
**Closed-loop simulation platform and neural network architecture for the Bioinspired BMI (B-BMI)**. **(A)** Closed-loop simulation platform for the B-BMI. The signal synthesizer (Synt-A) generates Poisson-distributed (inverted) pulse signals to simulate extracellular recordings from 18 primary motor cortex (M1) neurons and provides inputs for 18 analog channels of the DAQ device. SNN simulation tasks run the B-BMI by simulating the MSNs, which receive synapses from M1 neurons of Synt-A. A button press provides external input for the TTL control module to initiate a trial. Synt-A receives inputs from TTL control module to adjust the firing rates of the M1 neurons according to the selected target or the out-of-trial (baseline) status. **(B)** Tuning map of the M1 neurons of Synt-A (B, baseline; L, left; R, right direction) and neural network architecture for the B-BMI in closed-loop simulations. Red/green lines indicate synaptic connections between M1 neurons and left/right action MSNs.

In the closed-loop simulation paradigm, the MSNs of the B-BMI are simulated by the SNN simulation tasks of the BNDE and extracellular recordings from primary motor cortex (M1) neurons are simulated by the neural signal synthesizer. The synthesizer, implemented using a microcontroller (Microchip PIC18F4520), provides simulated neural signals to the analog input channels of the DAQ hardware of the BNDE through its output pins (Figure 7A). Each output pin of the synthesizer is associated with a synthetic M1 neuron and when a synthetic neuron generates a spike, the corresponding pin of the microcontroller produces an inverted 100-μs-duration pulse. As in previous studies in which closed-loop simulations were utilized for development of reinforcement learning-based BMI control algorithms (Mahmoudi and Sanchez, 2011; Mahmoudi et al., 2013), the synthetic neurons were created to reproduce the directional tuning properties of real M1 neurons. In the synthesizer, there are three cortical neuronal ensembles, each of which consists of six M1 neurons (Figure 7B). The neurons of the first ensemble are tuned to the “left” and the ones belonging to the second ensemble are tuned to the “right” direction. Additionally, the neurons of the remaining ensemble are tuned to “no direction” as uncorrelated neurons have been observed in *in vivo* neuroprosthetic control experiments (Sanchez et al., 2004; Wahnoun et al., 2006; Mahmoudi et al., 2013). The M1 neurons of the signal synthesizer are connected to the *in silico* MSNs through simulated synapses as explained in Figure 3; using the spike sorting task of the BNDE, the spikes sorted from the recordings are delivered to the *in silico* MSNs as synaptic events. As shown in Figure 7B, two-thirds of the synthetic neurons are connected to only one MSN and remaining one-third are connected to both MSNs. Note that, in this configuration, some neurons with directional tuning are connected to only one MSN, which represents the prosthetic action toward the opposite direction (e.g., neuron 2 in Figure 7B is tuned to left but connected only to right action MSN). Thus, the M1 neurons, regardless of directional tuning, are partially connected to the MSNs through simulated excitatory synapses.

**Figure 8:**
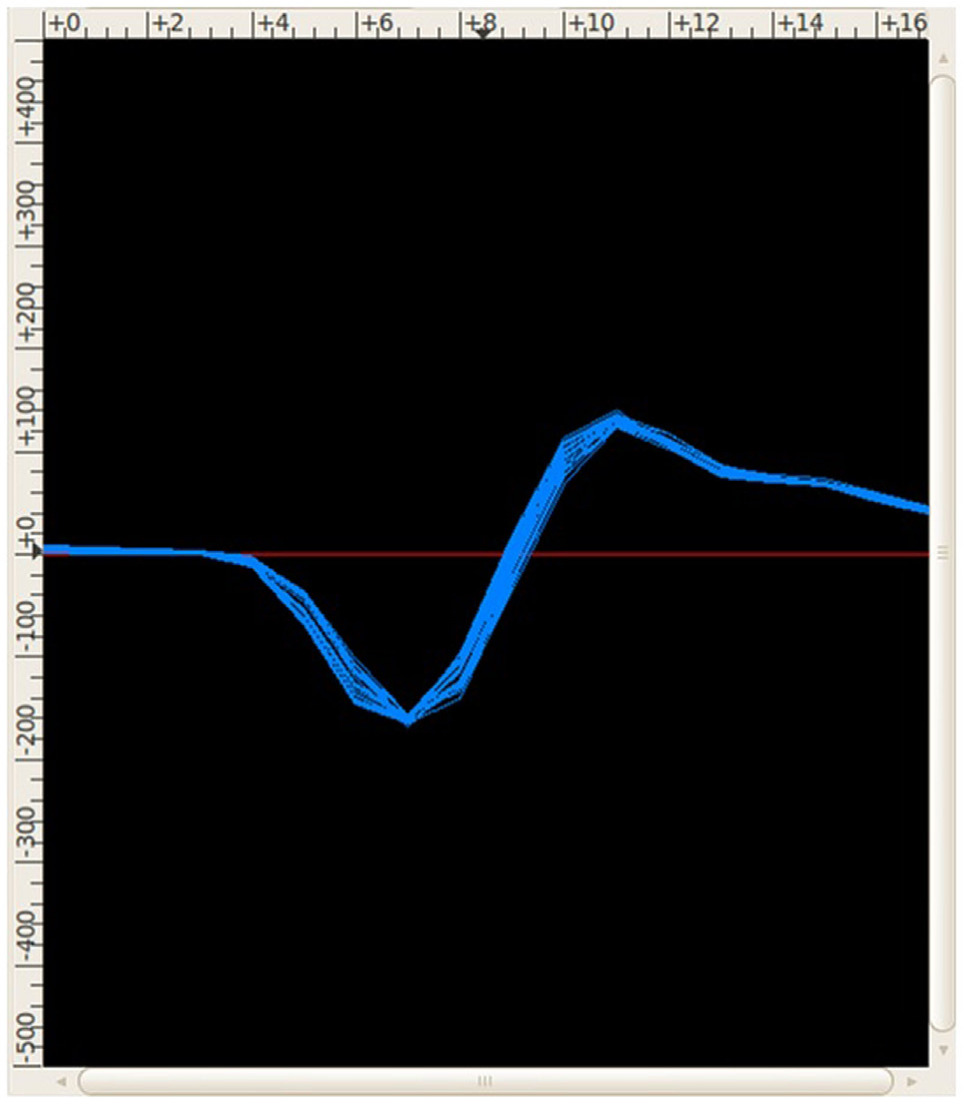
**The waveform of the spikes acquired from a channel of the neural signal synthesizer**. Snapshot was taken from the spike sorting GUI of the BNDE, which is also shown in Figure 4.

In order to simulate the directional tuning properties of the motor cortical neurons, we programed the neural signal synthesizer to produce Poisson-distributed spikes according to the tuning map shown in Figure 7B. Since the synthesizer was developed around a low computing power (8-bit) microcontroller, a *fast* 32-bit linear congruential pseudo-random generator (Press et al., 1992) was utilized to provide the random numbers for the Poisson-distributed spike generation process:

(13) x(s+1)=1664525x(s)+1013904223

where *x* is the generated 32-bit number, *s* is the number of the iteration and *x*(0) = 0. We studied the statistical properties of the present number generator and decided it was adequate for roughly simulating the firing rate properties of the motor cortex neurons in the development process of the B-BMI. In the closed-loop simulations, the Poisson-distributed spike generation process is run every 2 ms for each synthetic neuron. Throughout the simulations, the synthesizer continuously generates spikes according to the baseline firing rate estimates of the neurons. When a trial starts, it begins to apply the directional tuning properties of the neurons according to the selected target. The plot in Figure 8 is a snapshot from the spike sorting GUI of the BNDE while it was plotting the (digital band-pass filtered) waveforms of the neural spikes acquired from a channel of the signal synthesizer.

In the present closed-loop simulation paradigm, the spike sorting task of the BNDE is configured to perform sorting for multiple single-units for each recording channel as in an *in vivo* recording experiment. For each channel, “three single-units” are defined. The spike templates for the “first” single-units are generated by connecting the neural signal synthesizer to the input channels of the DAQ hardware. The templates for the “second” and “third” single-units are produced by applying two different sinusoidal waveforms to the input channels of the DAQ hardware. Since all spikes in the closed-loop simulations are generated by the neural signal synthesizer (Figure 7A), the spike sorting task sorts them into the “first” single-units, which correspond to M1 neurons of the synthesizer, and forwards the sorted spikes to the corresponding post-synaptic *in silico* MSNs (Figure 7B). In this paradigm, computational load of the spike sorting task for any detected spike is as much as the one in a real *in vivo* recording experiment, in which the spikes are sorted into up to three single-units for each channel, because it performs template matching by applying the likelihood function (Eq. 1) for “all three single-units” for each detected spike (see Spike Sorting via Template Matching).

**Figure 9:**
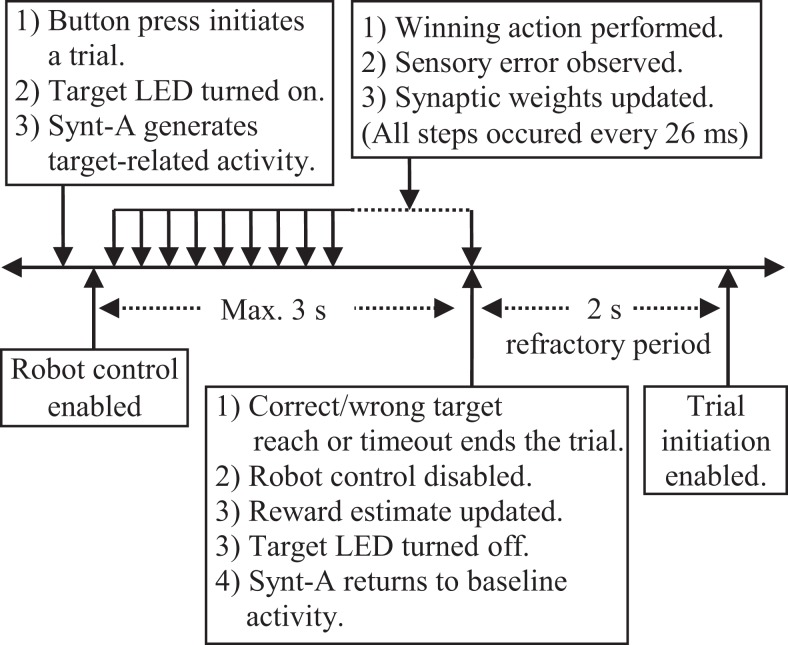
**Behavioral paradigm for the real-time closed-loop simulations**.

According to behavioral paradigm utilized in the real-time closed-loop simulations (Figure 9), each trial starts by an external digital input (i.e., button press), which is provided by the experimenter at arbitrary times. When the button is pressed for 26 ms, the DIO control task of the BNDE senses the external input through the TTL control module of the control hardware and sends a “trial start request” message to the experiment control task. After receiving the trial start request, the experiment control task selects a target (left or right) randomly and initiates a trial by delivering a message to the SNN simulation, the DIO control, and the prosthetic control tasks. The “trial start” message also includes the information regarding the selected target side (i.e., left or right). Thence, (1) the SNN simulation task, implementing the B-BMI, determines the value of the positive reward estimate $\left({\bar{R}}_{k}\right)$ corresponding to the selected target to apply Eq. 7 throughout the trial, (2) the prosthetic control task sets the Cartesian coordinates of the selected target to sense whether the target is acquired during a trial, (3) the DIO control task commands the TTL control module to turn the target LED on (Figure 6) and provides an input for the neural signal synthesizer. According to this input from the TTL control module, the neural signal synthesizer starts to generate the neural activity pattern related to the selected target (Figure 7B).

Forty milliseconds after a trial initiates, the prosthetic control task starts to periodically handle the spike events received from the SNN simulation tasks. Every 26 ms, it calculates the spike counts of the MSNs with a sliding 104-ms time window, selects the neuron with the highest spike count as the winning neuron and applies the prosthetic action corresponding to the winning neuron by delivering a command to the servo control module of the control hardware. This command rotates the (digital) base servomotor of the robotic arm −1°/+1° for the winning left/right actions or keeps it stationary in case of an equality among the spike count of the MSNs. When the spike counts of both MSNs are equal to 0, it also keeps the robotic arm stationary. Six milliseconds after delivering the pulse width command, the prosthetic control task receives the angle values of the joints through the servo control module and determines the Cartesian position of the tip of the arm by forward kinematics. If the tip of the robot moves toward the selected target in the last movement step (26 ms), the prosthetic control task sends a positive sensory error [*S*(*t*) = 1] message to the SNN simulation task. If not, it delivers a negative sensory error (−1). Based on the sensory error messages received from the prosthetic control task, the SNN simulation tasks periodically (every 26 ms) update the weights of the plastic synapses by applying Eq. 7.

Based on the Cartesian position of the tip of the robotic arm, the prosthetic control task periodically checks if the correct or wrong target is reached within the maximum trial duration (i.e., 3 s). If the correct (selected) target is reached, the prosthetic control task sends a “reward request” message to the ­experiment control task. If the wrong (opposite) target is reached by the robotic arm, a “punishment request” message is delivered to the experiment control task. By receiving such a request message, the experiment control task ends a trial by sending a message to the SNN simulation, DIO control, and prosthetic control tasks. The message to the SNN simulation tasks includes the information related to correct or wrong target reach so that it can update the positive reward estimate for the selected target, ${\bar{R}}_{k}$, by applying Eq. 10. The message to the DIO control task cancels the inputs to the target LED and the neural signal synthesizer. Thus, the synthesizer starts to generate the baseline spiking activity for the simulated cortical neurons. Finally, by receiving the “trial end message” from the experiment control task, the prosthetic control task directs the robotic arm back to its default position in the middle of the targets to prepare it to be used in the next trial.

In addition to the trial end messages, a trial is also terminated by the experiment control task when it is not completed by a correct or wrong target reach event within the maximum trial duration. When there appears a trial timeout, the experiment control task sends the “trial end message” to the downstream modules as in wrong target reach case. Thus, the positive reward estimate for the selected target, ${\bar{R}}_{k}$, is decreased, DIO control task cancels the input to the neural signal synthesizer and the prosthetic control task directs the robotic arm back to the default position. At the end of each trial, a refractory period of 2 s is applied to allow the robotic arm to reach its default position and to let the data writing processes to create new data folders for the recordings related to the next trial.

Throughout the closed-loop simulations, i.e., during the trials and inter-trial periods, the spike sorting task is always enabled to extract the spike events from the acquired data using the methods explained in Section “Real-Time Biological/*In Silico* Neuronal Interaction in the Hybrid Neural Network” and schedule synaptic events for the post-synaptic *in silico* neurons. Additionally, throughout the closed-loop simulations, the SNN simulation tasks evaluate the dynamics of the *in silico* cells and deliver the generated spike events to the prosthetic control task with a transmission delay of 3 ms (Figure 3). The prosthetic control task sorts the incoming events in the time domain and processes them only when the robotic control is enabled after a trial initiation.

For the simulation of the SNN, Parker–Sochacki (PS) method (Parker and Sochacki, 2000) is applied with the techniques presented by Stewart and Bair (2009) so that full-double precision accuracy is achieved in the numerical integrations. Whenever the SNN simulation tasks are triggered by timer interrupts, they perform numerical integrations for the differential equations describing the synaptic interactions and neuronal dynamics in the system (Eqs 2–5). For the integrations, the global step size is set to 250 μs. Prior to realizing integration for a global time step, the incoming synaptic events are sorted in the time domain. As PS method allows, the global integration step size is split into local substeps separated by the incoming synaptic events (if there is any) and integration for a global step size is realized through a single 250-μs-step or multiple substeps, accordingly. Whenever a spike is generated by a neuron, an event is scheduled for the post-synaptic neuron and the prosthetic control task (Figure 3) with nanosecond precision, which is the precision of the system time provided by RTAI.

### Stress test methods

**Figure 10:**
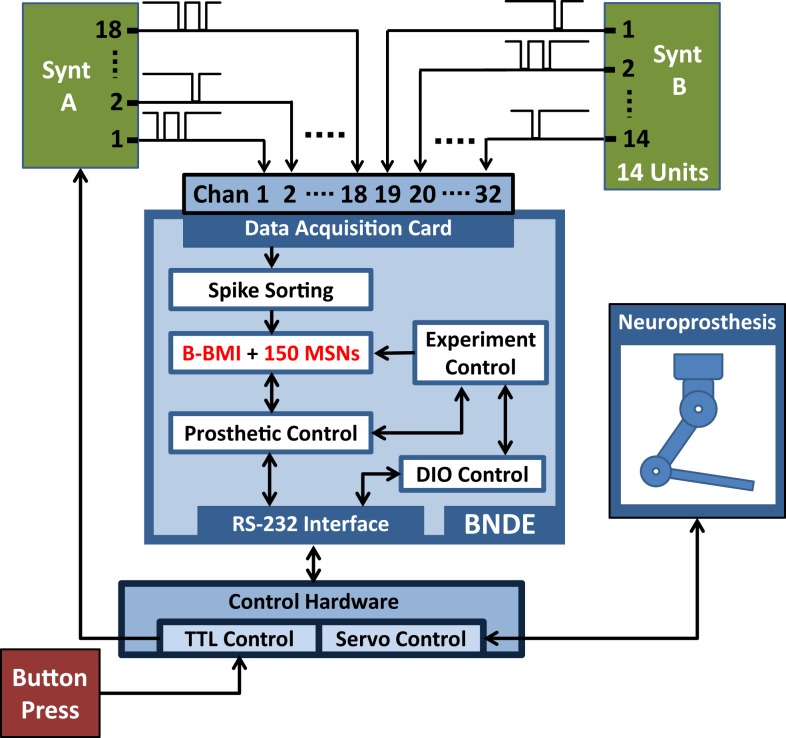
**The stress test platform**. For the stress test, an additional 14 channel neural signal synthesizer (Synt-B) and 150 MSNs are added into the closed-loop simulation paradigm shown in Figure 7A.

In order to evaluate the real-time computational capacity of the BNDE for its future uses in the design of more advanced SNN-based BMI controllers, which utilize a higher number of neurons or variables, we developed and implemented a stress test paradigm. In this paradigm, we utilized the same closed-loop simulation setup and the behavioral paradigm as the one mentioned in Section “Real-Time Closed-Loop Simulation Methods.” Additionally, we inserted 150 MSNs into the SNN and connected an extra neural signal synthesizer (Synt-B) to the remaining 14 channels of the DAQ board (Figure 10). However, these additional neurons and the signal synthesizer had no function in neuroprosthetic control; they were added into the closed-loop simulation platform only for stressing the hardware and software resources of the BNDE while the B-BMI was *learning* the control of the neuroprosthesis. In this scenario, the additional MSNs received inputs both from Synt-A and Synt-B through probabilistically connected excitatory synapses (connection probability = 0.66, the synaptic delays were selected from a uniform distribution ranging from 3 to 5 ms.). Moreover, these neurons were connected to each other through inhibitory synapses with a connection probability of 0.2; the transmission delays were selected from a uniform distribution between 2.5 and 3.0 ms. Consequently, each of the additional MSNs received approximately a total of 50 synapses from each other and from the signal synthesizers. Throughout the stress tests, each unit of the Synt-B continuously generated Poisson-distributed spikes with an estimated firing rate of 80 Hz independent from trial initiation or termination. The spike sorting task applied template matching algorithm (see Spike Sorting via Template Matching) for three single-units for each channel as in the closed-loop simulation paradigm.

## Results

### The B-BMI learning performance

Prior to starting the experiment to test the learning performance of the B-BMI, we determined the total weight of the excitatory synapses to the MSNs, *W* in Eq. 11, and the weight of the inhibitory synapses between the MSNs to provide a winner-take-all functionality in the network. While the Synt-A was generating baseline spike activity according to the tuning map as shown in Figure 7B, we empirically set the value of *W* to 110 nS to provide a baseline firing rate of approximately 2–5 Hz for the MSNs. The weight of the inhibitory synapses, which were non-plastic throughout the experiment, was set to a value of 40 nS to provide strong inhibition between the MSNs. The weight of the excitatory synapses to the MSNs was equal to each other at the beginning of the experiment and was continuously updated during the trials.

**Figure 11:**
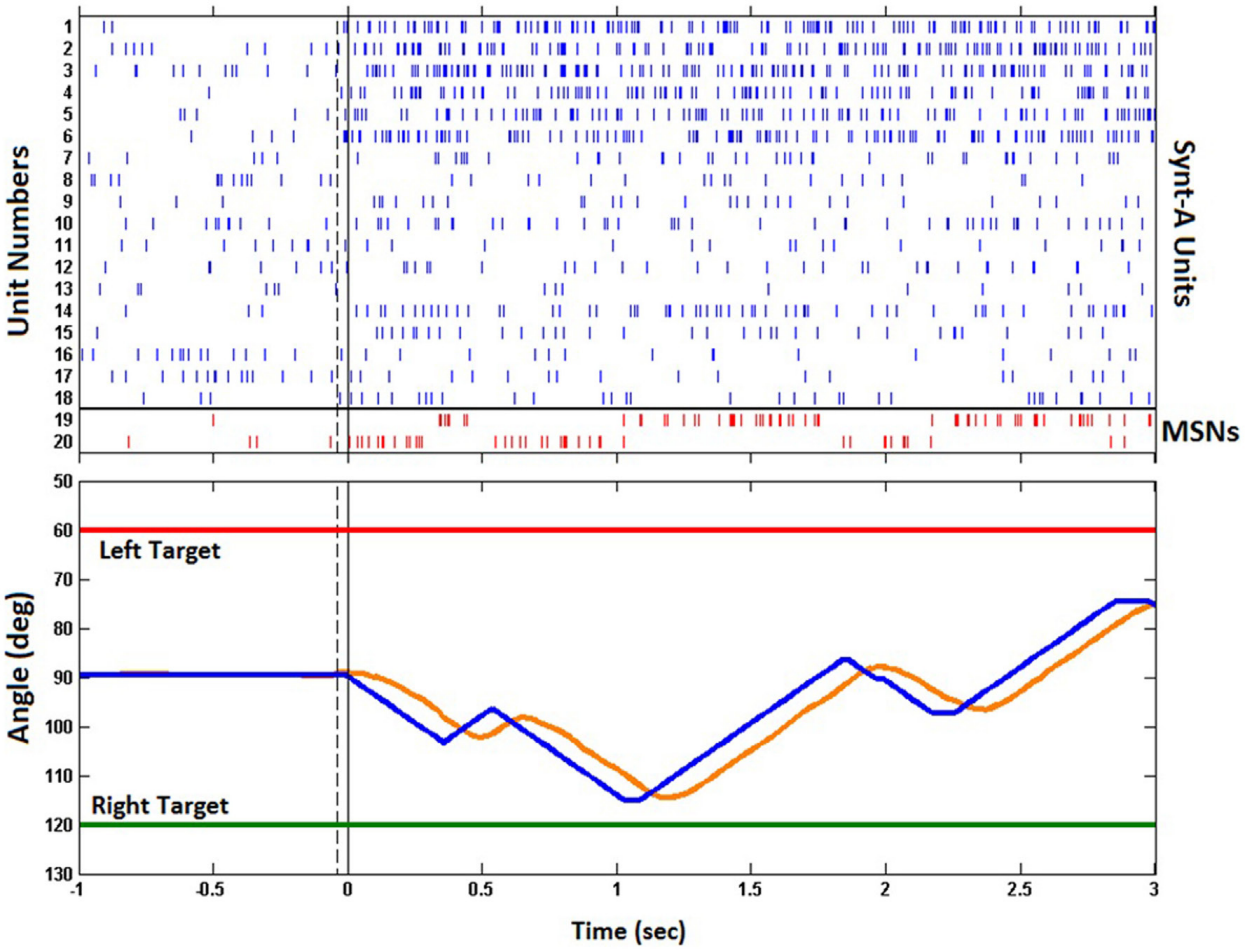
**Raster of the spikes and change in joint angle modified by the outputs of the MSNs during the first trial**. Upper plot shows the time of the spikes generated by Synt-A neurons (blue) for a selected left target and those by the MSNs (red) in response to the spike pattern generated by Synt-A. Note that the first six neurons of the Synt-A were tuned to left. The strong reciprocal inhibition between the MSNs can be realized through the activity pattern of the MSNs. Lower plot presents the trajectory of the robotic actuator. The blue trace indicates the joint angle values sent to the base servomotor of the robotic arm and the orange trace indicates its actual trajectory. Vertical dashed lines in both plots show the button press time to initiate a trial. Continuous black ones represent the time on which the robot control was enabled. Horizontal red/green lines present the position of the left/right targets in terms of the joint angle corresponding to the base servomotor of the robotic arm.

Figure 11 shows the raster plot of the spikes generated by the Synt-A and the MSNs during the first trial of the experiment. Prior to the trial, the Synt-A generated baseline spike activity. Upon initiation of the trial by a button press, the left target was selected by the experiment control task of the BNDE and the Synt-A started to produce the activity pattern for the left direction as it had been programed to imitate the tuning properties of motor cortex neurons (Figure 7B). The synaptic weights of the excitatory synapses to the MSNs were updated based on the sensory error extracted every 26 ms from the one-dimensional movements of the robotic arm. The trial was not rewarded since the robotic arm did not acquire the target within the maximum trial length of 3 s.

**Figure 12:**
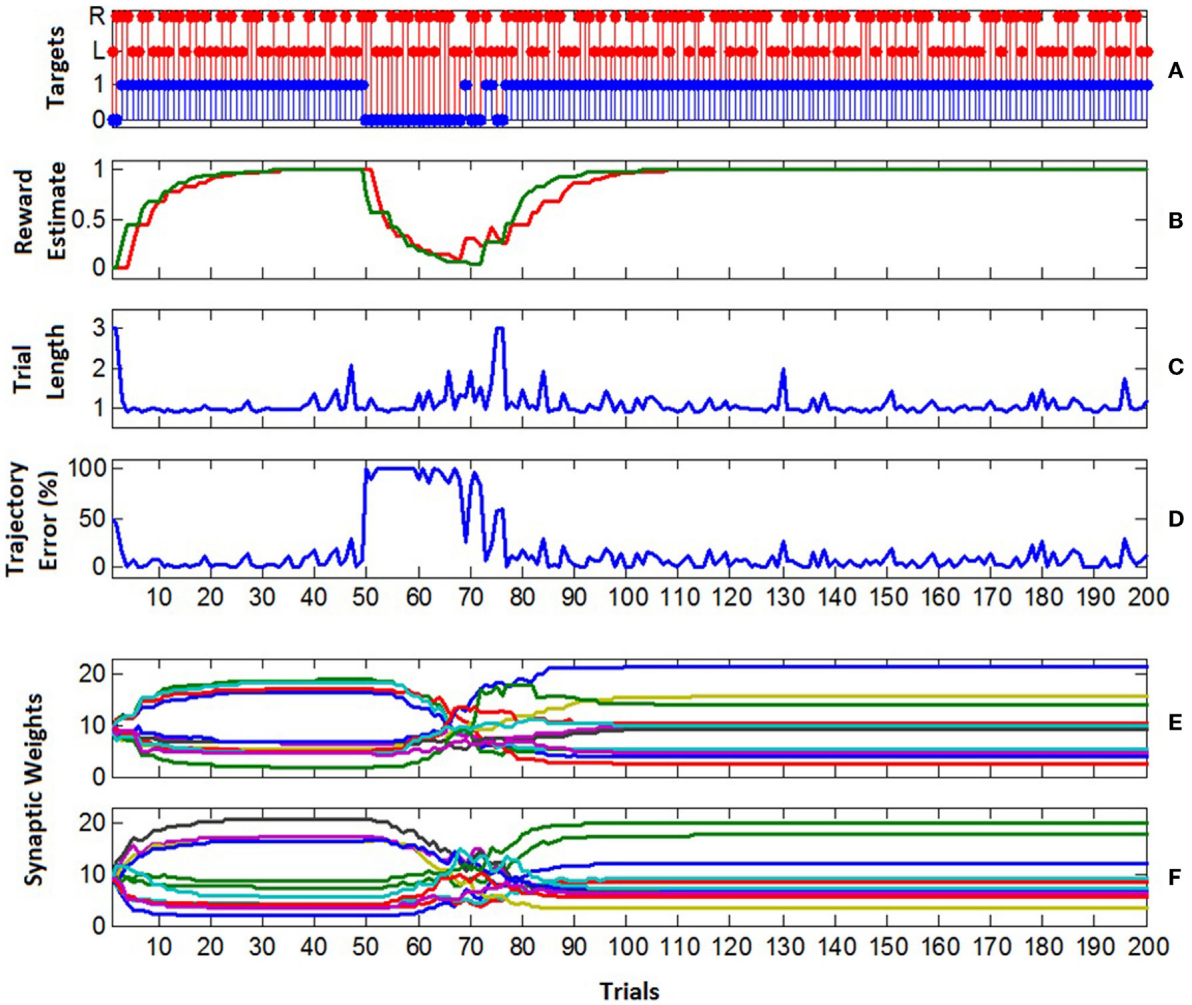
**Learning performance of the B-BMI**. **(A)** Target reach performance. The selected target for each trial is represented by red stems (L for left trial and R for right trial) and the blue stems shows if the target was acquired (1) or not (0) within maximum trial length. **(B)** The positive reward (successful target reach) estimate related to each target at the end of the trials (red for left, green for right target reach-related positive reward estimate). **(C)** The length of the trials. The wrong or correct target reach ends the trial before its maximum allowed length (3 s). **(D)** Trajectory error. The percent of the robotic actions, that does “not” direct robotic arm toward the selected target. The robotic actions are selected every 26 ms according to the spike count of the MSNs. Three actions were available: left, right and stationary. **(E,F)** The weight of the excitatory synapses of the left **(E)** and right **(F)** MSNs at the beginning of each trial. Convergence in synaptic weights was achieved at around trial 40 by updating the synaptic weights. At trial 50, the tuning map of the motor cortex units was reversed. At trial 77, 100% target reach accuracy was regained for the reversed tuning map.

The learning performance of the B-BMI is shown in Figure 12. As the control algorithm was naïve at the beginning of the experiment, the excitatory synaptic weights of the MSNs were set to be equal to each other and the positive reward (successful target reach) estimate for each target (${\bar{R}}_{k}$ in Eq. 9) was set to 0. In the first trial, the target was not achieved within the maximum trial length of 3 s as shown in Figures 11 and 12C. Thus, the trial was ended by a timeout and the positive reward estimate value $\left({\bar{R}}_{k}\right)$ for the selected left target stayed at 0 at the end of the first trial (Figure 12B). In this trial, approximately 50% of the pulse width commands, which are evaluated by the prosthetic control task and delivered to the control hardware every 26 ms, were not for directing the robotic arm toward selected left target. In other words, approximately 50% of the delivered commands were to keep it stationary or to direct it toward right. In this configuration, depending on the learning rate (0.02), perfect target reach accuracy was achieved after two unsuccessful trials. As the number of trials ending with a successful target reach increases, the positive reward estimate for each target climbed to “1.” At around trial 40, the excitatory synaptic weights of the MSNs converged at around trial 40 as shown in Figures 12E,F. As shown in Figures 12C,D, when the trajectory error is around 0%, the length of a trial was around 1 s.

In order to test the generalization performance of the B-BMI, at trial 50, we reversed the directional tuning map of the motor cortex units; i.e., the tuning property of the units for left direction was switched to be generated for the right direction, and vice versa. In addition, the firing rate estimate of the units for the baseline activity remained the same. In this reversal learning paradigm, from the view of the MSNs, the task was to update the synaptic weights effectively to regain the perfect target reach accuracy. From Figure 12D, we can see dramatic increase in trajectory error in trial 50. In this trial, the trajectory error was 100% and the trial length was around 1 s since the robotic arm directly reached the wrong target. As the target reach accuracy of the control algorithm decreased, the reward estimate for the targets also diminished. At trial 69, target reach accuracy of the algorithm started to increase again and corresponding reward estimate value for the selected target was updated accordingly (Figure 12B). As the reward estimate for the targets rose, the trajectory error declined. At around trial 120, the synaptic weights of the MSNs converged to their final values and stayed there until the end of the experiment (Figures 12E,F). After convergence (between trial 120 and 200), the average trajectory error percentage was approximately 5.9%.

### Performance of the BNDE

In order to evaluate the performance of the BNDE, we ran two test cases: First, we ran the B-BMI using the closed-loop simulation methods explained in Section “Real-Time Closed-Loop Simulation Methods” (“only B-BMI paradigm”). Second, we ran the stress test paradigm explained in Section “Stress Test Methods” (“B-BMI + 150 MSNs”). Each test case was run for 2 h while online visualization and data recording tools of the BNDE were enabled. For each test case, 500 trials were performed on the system. Additionally, at trial 50 of each test, the tuning map for the motor cortex units were reversed as mentioned in the previous section.

At the end of both test cases, the SNN simulation task and the prosthetic control task did not miss any spike event generated by the neural signal synthesizers (Synt-A&B) and the SNN simulation tasks; all spike events extracted by the spike sorting task were processed by the SNN simulation tasks on their scheduled time and prosthetic control task handled all spike events in the corresponding time bin (Figure 3). During these tests, we observed execution times of the RT tasks and deviations in their periods (i.e., jitters) in order to evaluate the real-time performance of the system. Based on measurements through 100,000 consecutive execution cycles, average jitter for the tasks triggered by the timer interrupts of the system (i.e., all tasks except for spike sorting task) was <1 μs, with a maximum of 20–25 μs. For the spike sorting task, which was triggered by the interrupts of the DAQ hardware, the average jitter was approximately 9 μs, with a maximum of 110–120 μs. In the BNDE, spikes are timestamped according to the system time at which the spike sorting task is triggered to execute (see Real-Time Biological/*In Silico* Neuronal Interaction in the Hybrid Neural Network). Therefore, these jitters could lead to a negligible error in spike timestamping process. Thanks to the clock synchronization algorithm explained in Section “Real-Time Biological/*In Silico* Neuronal Interaction in the Hybrid Neural Network,” the error in spike timestamping is even less than these jitter levels.

Table 2 presents the average and maximum execution times of the RT tasks during both tests based on 100,000 execution cycles. From the table, we can see that increasing the number of the MSNs for the stress test, as expected, also increased the execution time of the SNN simulation tasks. By addition of the Synt-B into the simulation platform for the stress test paradigm, the average execution time for the spike sorting task also increased due to operation of template matching algorithm for additional 14 DAQ channels. However, the execution time for other tasks was not significantly affected by such increases since they were run on different cores of the CPU.

**Table 2:** **Execution times of the RT tasks during running only the B-BMI and the stress test**.

	Only B-BMI		Stress test (B-BMI **+** 150 MSNs)		Task period (**μ**s)
RT task	Average (**μ**s)	Maximum (**μ**s)	Average (**μ**s)	Maximum (**μ**s)	
Spike sorting	59	145	71	188	512
SNN simulation 0	7	22	767	1614	2000
SNN simulation 1	7	20	770	1578	2000
Prosthetic control	5	55	6	61	2000
Experiment control	2	31	2	28	2000
DIO control	3	15	3	15	2000

During running only the B-BMI and the stress test paradigm, the BNDE recorded (1) the timestamps of the spikes generated by both the biological and *in silico* neurons, (2) the input and output events related DIO control task, (3) the pulse width commands sent to the servo control module, (4) joint angle values received from the servo control module, and (5) the statistics related to the experiments. At the end of the performance test for the only B-BMI case, the BNDE recorded 48 MB of data. In the case of the stress test, as the number of the spiking units was increased by an addition of a neural signal synthesizer (Synt-B) and 150 MSNs to the SNN simulator, the BNDE recorded 1.18 GB of data in 2 h. During the stress tests, the average spiking activity per simulated MSN was approximately 41 Hz.

**Figure 13:**
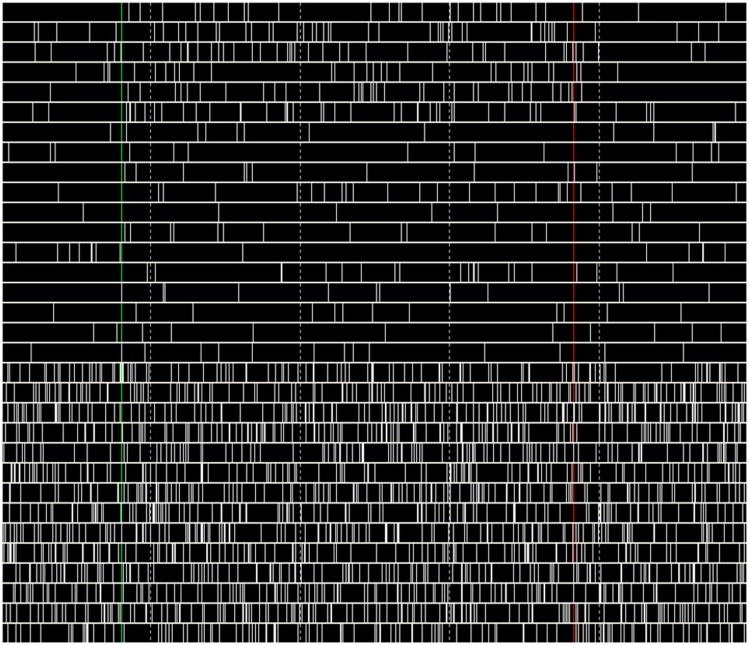
**Raster plot of the spikes generated by Synt-A&B during 462nd trial of the stress test**. The first six rows present the tuned activity of the neurons simulated by the Synt-A. Last 14 rows reflect the activity pattern of the high-frequency spiking neurons of the Synt-B. The time interval between the vertical dashed lines is 300 ms.

**Figure 14:**
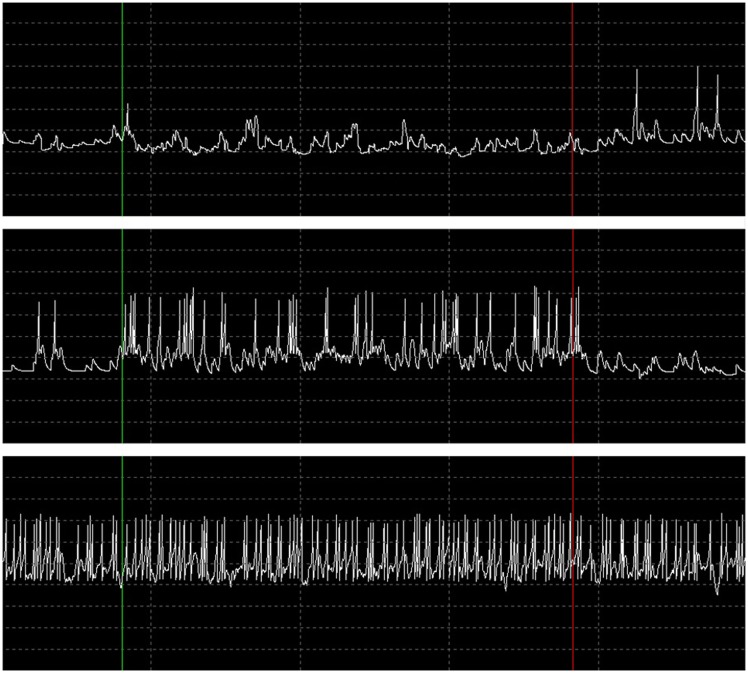
**Dynamics of the MSNs during 462nd trial of the stress test in response to the spike events presented in Figure 13**. When the trial was started and the robot control was enabled (vertical green line), the MSN corresponding to right action was activated (*switched mostly to up-state*) by the increasing activity of the simulated motor cortex units (graph in the middle) and suppressed the activity of the left action MSN (uppermost graph) through the inhibitory synapse. At the end of the trial by successful target reach (vertical red line), the MSNs returned to their baseline activities with the decrease in the activities of the pre-synaptic motor cortex units. The bottom graph represents the neuronal dynamics of one of the MSNs, which were added into SNN for the stress test paradigm. The time interval between the vertical dashed lines is 300 ms and the voltage difference between the horizontal dashed lines is 30 mV.

During the performance tests for running only the B-BMI and the stress test paradigm, we were able to monitor the plot of the dynamics of the MSNs and raster of the spike events generated by the neural signal synthesizers (Synt A&B). Figure 13 shows a snapshot from the GUI of the BNDE while it was plotting the raster of the spikes online during the 462nd trial of the stress test. Additionally, Figure 14 shows the snapshot of the GUI while plotting the dynamics of three manually selected MSNs during the same trial. The uppermost graph in Figure 14 illustrates the membrane potential dynamics of the MSN, which corresponds to the left prosthetic action and the graph in the middle presents those of the MSN corresponding to the right action. Finally, the bottom graph shows the high-frequency spiking activity of one of the 150 MSNs added into the system for the stress tests. The green vertical lines in Figures 13 and 14 mark the timepoint on which the control of the robotic actuator was enabled and the red lines indicate the timepoint on which the trial was ended by acquisition of the right target. Even though the GUIs are capable of plotting 3-s history of the raster of the spikes and the neuronal dynamics, we took the snapshot of the same 1.5 s portion of the visualized part in order to provide a higher resolution image here.

## Discussion

### The BNDE

In the present work, one of our goals was to implement a platform capable of creating simulated synaptic connections from extracellularly recorded neurons to model neurons for development of SNN-based BMI controllers. Since the software-based SNN simulations provide a flexible method for investigating the behavior of the neuronal circuits, we preferred to develop this platform around a desktop PC. In order to satisfy the strict timing constraints of the real-time SNN simulations and biological/*in silico* neuronal interactions on PC, we used RTAI, a real-time extension for Linux operating system. Utilization of RTAI provided several benefits in establishment process of the BNDE. First, it enabled development of real-time applications, which are equipped with powerful GUIs capable of live visualization of the spiking activity of the biological and *in silico* neurons (Figures 13 and 14). Second, by the support of the COMEDI drivers, the RTAI provides deterministic response to the interrupts of a DAQ device. Thus, the neural data acquired from the brain (Figure 4) or neural signals synthesizers (Figure 8) could be processed in real-time. Third, since the RTAI provides serial port drivers, it became possible to perform the control of a robotic actuator according to the outputs of a SNN-based BMI controller, which was trained using the position-related feedbacks received from the actuator. Finally, the use of the open-source software provided by RTAI and COMEDI projects significantly decreased the costs in establishment of the BNDE. Even though there are advanced software projects such as RTXI^4^ and RELACS^5^, which are based on RTAI and COMEDI for dynamic patch clamp studies, real-time neural DAQ and stimulation, there is no work which specifically targets creating hybrid neural networks for control of motor neuroprostheses. In this work, we demonstrate how the BNDE establishes simulated synaptic connections from extracellularly recorded motor cortex neurons to model neurons for neuroprosthetic control. We also present how the BNDE efficiently utilizes multiple CPU cores for computationally intensive real-time SNN simulations. The scheduled event buffering mechanisms (see Real-Time Biological/*In Silico* Neuronal Interaction in the Hybrid Neural Network), which are specifically designed to avoid use of mutual exclusion locks, allow efficient utilization of computational resources of the system. The tasks running on different CPU cores can simultaneously deliver events to the target tasks without waiting for each other to unlock any mutual exclusion. Moreover, based on utilization of RTAI, the BNDE can also be used for interfacing a SNN for the control of a three degree-of-freedom robotic arm without requiring a DAQ device.

The other motivation for this study was to evaluate the real-time performance of the BNDE prior to its use in animal experiments. In order to test all its implemented software components, we connected an external, hardware-based neural signal synthesizer to the analog input channels of the DAQ hardware and developed the B-BMI controller using the simulated cortical inputs provided by this signal synthesizer (Figure 7A). Additionally, we interfaced the B-BMI with a robotic arm operating in real-world. Using the behavioral paradigm presented in Figure 9, which involved external binary inputs (e.g., button press) to initiate neuroprosthetic control trials and outputs to indicate the position of the targets (e.g., LED targets), the system learned the control of the robotic arm for a two-target reaching task in one-dimensional space. In these simulations, the spike sorting task of the BNDE sorted the spikes by applying the template matching algorithm for multiple single-units as in *in vivo* recording experiments (see Real-Time Closed-Loop Simulation Methods). In addition to the real-time closed-loop simulations with neural signal synthesizers, the classification performance of the spike sorting utility of the system was also validated by performing *in vivo* neural recordings from the rat motor cortex (Figure 4). These performance profiles indicate that the BNDE can be used in future *in vivo* neuroprosthetic control experiments.

Since we plan to utilize the BNDE in the long run for development of BMI controllers, which are based on larger scale SNNs or larger number of neuron/synapse model parameters, we examined its performance by a stress test paradigm involving simulation of 150 neurons, which received dense synaptic connections from each other and the synthetic units of the neural signal synthesizers (see Stress Test Methods). Throughout the stress test, the BNDE was capable of manipulation of the neuroprosthesis using the B-BMI while simulating additional 150 MSNs without missing any spike event generated by the neural signal synthesizers or the simulated MSNs.

Since the COMEDI project provides drivers for a variety of DAQ boards, the BNDE can be implemented using products of other vendors as long as the drivers for those boards support adequate sampling frequency for spike sorting. In addition, the DAQ boards should be capable of being programed to deliver on-board buffered data to the system memory periodically and generate an interrupt at the end of data delivery process so that the spike sorting task can be triggered in response to the interrupts. Moreover, the DAQ device, which is to be utilized, should be capable of buffering an adequate amount of samples into its on-board memory and deliver them to the system memory at an appropriate frequency; if the data transmission period, and the resultant interrupt generation period, for the DAQ device is too low, then the GUIs of the system might be unusable due to consumption of the system resources for processing these excessively frequent high-priority hardware interrupts. We determined an appropriate period (512 μs corresponding to 16 scans) for the DAQ interrupt generation in the BNDE so that all the components of the system worked smoothly.

In the BNDE, memory allocation for the data required for spike sorting and spike waveform displaying is realized during compile time by setting a software configuration parameter. This parameter represents the maximum number of the single-units, which can be sorted from a DAQ channel. Therefore, the number of the single-units, which can be defined per channel, can be easily increased by changing this parameter during compile time as long as the spike sorting GUI has space to display the spike waveforms related to each single-unit (Figure 4). Based on the template matching algorithm used (see Spike Sorting via Template Matching), spike sorting is realized by running Eq. 1 for all defined single-units for each DAQ channel. Consequently, execution time of the spike sorting task increases almost linearly proportionally to the number of single-units for which spike sorting is activated. Table 2 presents the execution time of the spike sorting task during closed-loop simulations and the stress test. From this table, a baseline for execution time of the spike sorting task can be perceived. This baseline execution time is related to data buffering, digital filtering, and up-sampling processes, which are performed by the spike sorting task for all 32 DAQ channels even if there is no spike sorting is activated for any single-unit.

We used a quad-core PC in the present implementation of the BNDE. As the software architecture of the BNDE and RTAI allows the SNN to be simulated by multiple RT tasks assigned to different cores of the CPU, the number of neurons, which can be simulated by the system, can be improved by utilization of CPUs consisting of a higher number of cores. Additionally, in the present study, we utilized PS integration method with double precision accuracy in order to maximize the accuracy in the SNN simulations. By sacrificing the accuracy in numerical integrations, a higher number of neurons can be simulated in the system using well-known integration methods, such as Euler and Runge-Kutta methods (Stewart and Bair, 2009).

Using the serial port driver of RTAI, which guarantees real-time serial communication, the prosthetic control task delivers pulse width command messages to the control hardware every 26 ms with low latency. Each pulse width command message consists of 10 bytes and includes the pulse width commands for all three servo motors of the robotic arm used. In the real-time simulations presented in this work, one of the pulse width commands delivered to the control hardware was for driving the base servomotor of the robotic arm for left/right movements and the other two pulse width commands were for keeping the remaining servo motors stationary. In the present system design, whenever the control hardware receives a pulse width command message from the prosthetic control task, it immediately applies pulses for the all three servo motors within maximum 6 ms. From Figure 11, we can see the inertia of the robotic arm from the difference between the delivered pulse width commands and actual trajectory of the robotic arm. These performance results indicate that the present system can provide suitable feedback for the BMI user during *in vivo* experiments. The communication protocol used between the prosthetic control task and the control hardware technically supports three-dimensional control of the present robotic arm since it can deliver commands to control hardware for all three servo motors and read the value of their joint angles every 26 ms.

Since the RTAI and COMEDI libraries enable utilization of multiple DAQ devices on a single PC, additional DAQ devices can be inserted into the system so that the number of units isolated from the neural recordings can be increased to provide a higher number of synaptic connections to the model neurons from the real neurons. The number of DAQ cards, which can be inserted into the system, is limited by the PCI/PCI Express ports of the system used. Since the spiking model neurons are *event-driven computing units*, their utilization in neuroprosthetic design can enable development of bidirectional BMIs, which hold potential for substituting malfunctioning brain circuits (Berger et al., 2012; Hogri et al., 2015). RTAI and COMEDI libraries utilized in development of the BNDE also support hard real-time control of the digital outputs of DAQ devices. Therefore, the BNDE can be improved further to control some optical (Han et al., 2009) or electrical stimulation (Venkatraman and Carmena, 2011) devices through the digital output channels of DAQ devices. The spike outputs of the SNN simulation task of the BNDE could be used to stimulate the brain tissue in a bidirectional BMI control paradigm.

### The B-BMI

A B-BMI controller is proposed using real-time closed-loop simulations. It utilizes two model MSNs, each of which represents one of two prosthetic actions and competes with the other through strong inhibitory synapses. The total weights of the excitatory synapses to each MSN (*W* in Eq. 11) and the weights of the inhibitory synapses between the MSNs are adjusted to realize a winner-take-all operation in the system. It would be interesting to determine the type of Izhikevich neuron, which provides the best speed for switching between the selected actions and the best noise robustness for a selected action in a winner-take-all type classification operation. This topic is out of the scope of the present work, we intend to investigate this topic in the near future.

Learning (or adaptation) in the B-BMI was achieved by reward-modulated spike timing-dependent plasticity. The excitatory synapses leading to correlated pre- and post-synaptic activity were tagged using eligibility traces. A positive global reward signal, which may characterize a phasic increase in dopamine concentration, led to LTP in the eligibility-tagged synapses (Reynolds et al., 2001; Schultz, 2001). In contrast, a negatively signed global reward signal, which may represent a phasic depression in dopamine concentration, caused LTD in the tagged synapses (Reynolds and Wickens, 2002). In the present paradigm, the sign of the global reward signal [*r*(*t*)] was determined by the sensory error [*S*(*t*)], which was extracted from the movements of the robotic arm. The robotic movements toward the currently selected target led to an increase in reward expectancy and a positive global reward signal, and opposite-direction movements triggered a negative reward signal. Additionally, reaching behavior toward each target was treated as a different task to be learned and the system held a separate positive reward (successful target reach) estimate value $\left({\bar{R}}_{k}\right)$ for each task (Frémaux et al., 2010). As the reward estimates increased by acquisition of the correct targets in consecutive trials, the magnitude of the global reward signal [*r*(*t*)] was degraded (Tobler et al., 2005) so that the synaptic weights were automatically stabilized when perfect target reach accuracy was ensured for each target (Figure 12). Learning speed and convergence characteristics of the synaptic weights in the present controller can be modified by changing the learning rate in Eq. 8 and the reward estimate averaging window size in Eq. 10.

The B-BMI controller always aims to maximize the positive reward (successful target reach) estimate value $\left({\bar{R}}_{k}\right)$ for any selected target. To this end, when the positive reward estimate value, in a trial, is “<1” for the selected target, the controller updates the weights of the excitatory synapses of the MSNs *during* reaching movements. During the (correct) movements toward the selected target, the weights of all tagged synapses are increased. In contrast, during the movements toward the wrong target, the weights of the tagged synapses are decreased. Therefore, the probability of selecting the correct prosthetic movement for a given motor cortex activity pattern is increased by trial-and-error, by reinforcement learning (Chadderdon et al., 2012; Neymotin et al., 2013). When the value of positive reward estimate value $\left({\bar{R}}_{k}\right)$ reaches “1,” synaptic weight update automatically stops and the convergence in the system is achieved. Whenever the value of reward estimate $\left({\bar{R}}_{k}\right)$ becomes “<1” due to wrong target reach or trial timeout, the synaptic weights are re-updated in the system to maximize the value of ${\bar{R}}_{k}$ again.

In the present study, the units of the neural signal synthesizer (Synt-A) simulated the spiking behavior of motor cortical (excitatory) regular spiking neurons. During the inter-trial periods, the units generated baseline activity, which was characterized by low-frequency spike trains and, during the trials, the units with directional tuning increased their spiking activity according to selected target (Putrino et al., 2010) (Figure 7B). As it is possible to distinguish the (excitatory) regular spiking units from (inhibitory) fast spiking ones through the electrophysiological recordings (Fee et al., 1996; Putrino et al., 2010), we foresee that it will be possible to provide artificial excitatory synaptic inputs to the simulated MSNs from the cortical regular spiking units during future *in vivo* neuroprosthetic control studies. In addition, throughout the closed-loop simulations, the tuning map for the units of the neural signal synthesizer was static; the tuning properties of the units did not alter by experienced rewards. Since the motor cortex neurons have the capability to adapt their activity patterns for efficient control of neuroprostheses (Koralek et al., 2012; Arduin et al., 2014), we expect that cortical neuroplasticity will have a positive effect on the performance of the developed control algorithm (Ganguly et al., 2011). The learning rate in Eq. 8 determines the adaptation between the brain and the B-BMI. When it is decreased, the contribution of the brain, by neuroplasticity, is expected to be more pronounced in the neuroprosthetic control. In contrast, increases in the learning rate will have pronounced effect on the adaptability of the B-BMI. If the learning rate is set too high, then the co-adaptation between the brain and the B-BMI may not be accomplished.

## Conclusion

We present the BNDE as a practical platform for creating hybrid biological/*in silico* neural networks and developing neurally inspired neuroprosthetic systems. Additionally, we propose a novel BMI controller (the B-BMI), which was designed on the BNDE using real-time closed-loop simulations. Performance profiles of these simulations, involving a behavioral paradigm and an external neural signal synthesizer, not only show that the BNDE is capable of creating simulated synaptic connections from real neurons to *in silico* neurons during behavioral experiments but also present an important proof-of-concept for SNN-based neuroprosthetic control.

The proposed BMI controller, the B-BMI, is based on a hybrid neural network consisting of real motor cortical neurons and *in silico* MSNs. In this control paradigm, the MSNs are represented by Izhikevich’s biologically plausible model (Izhikevich, 2007b) and receive simulated synapses from motor cortical neurons. Adaptation of the present controller is realized by simulating a possible mechanism of dopamine-dependent synaptic plasticity; a reward signal, which may characterize phasic changes in dopamine concentration, is used to update the weights of the eligibility-tagged synapses. Future work will be aimed at studying the performance of the present BMI controller by realizing *in vivo* experiments and investigating the dopaminergic neuronal activity during neuroprosthetic learning.

The control architecture of the B-BMI is fundamentally distinct from those of conventional neuroprosthetic systems. Conceptually, most of the conventional systems utilize an input–output mathematical model, which maps motor cortical activity into user’s intended prosthetic actions. In these systems, a “spike binning” preprocess is also performed to provide cortical firing-rate inputs to the input–output model used and this preprocessing leads to loss in the information encoded by timing of the spikes. In contrast, in the present system, the control of the neuroprosthesis is realized in a more biologically plausible manner. Cortical spike events are directly forwarded to the model neurons through simulated synapses without spike binning and the neural information provided by spike timing is also used by the present BMI controller.

The BNDE provides a low-cost and extendible solution for development of novel BMI control algorithms, which utilize model neurons as neural information processors. It allows the neuroprosthetic designer to visualize the dynamics of the hybrid neural network online and manage the behavioral experiments through GUIs while time-critical spike sorting, real-time SNN simulation, and neuroprosthetic control are realized in the background. Since the BNDE has been developed on RTAI (a real-time extension for Linux); its software components can easily be modified for a wide variety of PC hardware platforms.

## Conflict of Interest Statement

The authors declare that the research was conducted in the absence of any commercial or financial relationships that could be construed as a potential conflict of interest.

## Footnotes

^1^www.rtai.org

^2^www.gtk.org

^3^www.comedi.org

^4^www.rtxi.org

^5^www.relacs.net
